# DNA Damage Response and Immune Defense: Links and Mechanisms

**DOI:** 10.3389/fgene.2016.00147

**Published:** 2016-08-09

**Authors:** Rania Nakad, Björn Schumacher

**Affiliations:** ^1^Institute for Genome Stability in Ageing and Disease, Medical Faculty, University of CologneCologne, Germany; ^2^Cologne Excellence Cluster for Cellular Stress Responses in Aging-Associated Diseases, Center for Molecular Medicine Cologne and Systems Biology of Ageing Cologne, University of CologneCologne, Germany

**Keywords:** DNA damage, DNA damage response (DDR), immune defense, chronic inflammation, tumourigenesis

## Abstract

DNA damage plays a causal role in numerous human pathologies including cancer, premature aging, and chronic inflammatory conditions. In response to genotoxic insults, the DNA damage response (DDR) orchestrates DNA damage checkpoint activation and facilitates the removal of DNA lesions. The DDR can also arouse the immune system by for example inducing the expression of antimicrobial peptides as well as ligands for receptors found on immune cells. The activation of immune signaling is triggered by different components of the DDR including DNA damage sensors, transducer kinases, and effectors. In this review, we describe recent advances on the understanding of the role of DDR in activating immune signaling. We highlight evidence gained into (i) which molecular and cellular pathways of DDR activate immune signaling, (ii) how DNA damage drives chronic inflammation, and (iii) how chronic inflammation causes DNA damage and pathology in humans.

## The DNA Damage Response (DDR)

The DNA damage response (DDR) is a complex signal transduction pathway that is required for preserving the genetic information encoded by DNA and for ensuring its accurate transmission through generations. Erroneously repaired DNA lesions can lead to mutations while unrepaired damage can result in cellular senescence or apoptosis (Welsh et al., 2004; Mendoza et al., 2013; Ciccia and Elledge, 2010). Dysregulation of DDR and repair systems can cause several human disorders that are associated with cancer susceptibility, accelerated aging, and developmental abnormalities (Pan et al., 2016).

The DDR is triggered by a wide variety of physico-chemical aberrations in the genome. Some DNA aberrations are caused by physiological processes such as base mismatches introduced during DNA replication and DNA strand breaks caused by malfunctioning activity of topoisomerase I and II (Jackson and Bartek, 2009). Lesions in the DNA can also arise from the release of reactive oxygen species (ROS) upon oxidative respiration or through redox-cycling events mediated by heavy metals (Valko et al., 2006). Other DNA damaging agents are ultraviolet light, ionizing radiation and a large variety of chemical agents (Hoeijmakers, 2009). Also replication stress resulting from oncogenic signaling can result in genome instability (Halazonetis et al., 2008). These endogenous and exogenous factors induce diverse lesions in the DNA such as nucleotide alterations (substitution, deletion, and insertion), bulky adducts, single-strand breaks (SSBs) and double-strand breaks (DSBs) (Rodriguez, 2011).

DNA damage recognition is the initial step of DNA damage repair mechanisms and involves a set of lesion-specific sensing molecules. Damage detection is followed by the recruitment of a set of transducers, which are composed of a number of protein kinases. Finally, different checkpoints and repair systems (effectors) including cell cycle regulators, nucleases, helicases, polymerases, ligases are involved in removing the damage, thus maintaining genome integrity (Pan et al., 2016).

In recent years, several lesion-specific repair mechanisms have been identified. Non-homologous end joining (NHEJ) and homologous recombination (HR) repair both remove DSBs through distinct mechanisms (West, 2003), single-strand break repair (SSBR) ligates nicked DNA strands (Caldecott, 2008), mismatch repair (MMR) restores errors that occurred during replication (Jiricny, 2006), base excision repair (BER) reverses oxidative base modifications (Lindahl and Barnes, 2000; Bauer et al., 2015), and nucleotide excision repair (NER) removes helix-distorting lesions (Hoeijmakers, 2009; Edifizi and Schumacher, 2015).

## The Immune System Responds to DNA Damage

Immune signaling in response to DNA was reported as early as 1963 when Isaacs et al. (1963) showed that mouse cells that had been infected with chick nucleic acid produced cytokines and interferons (IFNs). Ever since, significant progress has been made toward understanding the role of DNA in activating the immune system. As endogenous DNA in eukaryotes is stored in the nucleus, infectious foreign DNA enters the cytoplasm, where it is detected and responded upon by the host’s immune system. Bacterial DNA has been shown to activate the innate immune system and stimulate inflammatory responses [reviewed in Krieg (2002)]. Not only DNA, but also RNA injected by viruses into human cells induces the expression of IFN encoding genes (Ank et al., 2006). Intriguingly, not only viral and bacterial pathogen-associated molecular patterns (PAMPs) such as foreign DNA but also damaged endogenous DNA can trigger inflammatory gene expression. For instance, treatment of human cells with etoposide, an anticancer drug promoting dsDNA breaks by inhibiting the ability of topoisomers II to re-ligate cleaved DNA (Meresse et al., 2004; Baldwin and Osheroff, 2005), leads to the induction of IFN-stimulated genes, primarily IFN-α and IFN-λ genes (Brzostek, 2011). Innate immune responses to damaged endogenous DNA have been evidenced in various species including nematodes and fruit flies. Recent observations in the nematode *Caenorhabditis elegans* established that DNA damage in germ cells including meiotic DSBs triggers the worm’s innate immune response through ERK1/2 MAPK signaling (Ermolaeva et al., 2013). *C. elegans* lacks specialized immune cells or adaptive immunity but has an ancestral innate immune system that is activated in response to various pathogens through several immune cascades including the p38 and ERK MAPK pathway (Ermolaeva and Schumacher, 2014a). Exogenous and endogenous DNA damage in germ cells mediated a germline DNA damage-induced systemic stress resistance (GDISR) throughout the somatic tissues of the animal resulting in elevated resistance to heat and oxidative stress (Ermolaeva et al., 2013; Ermolaeva and Schumacher, 2014b).

The understanding of how DDR induces immune responses has remained a challenging question (Pateras et al., 2015; Ribezzo et al., 2016). How can cytoplasmic immune sensors detect nuclear DNA? How are nuclear DNA sensors linked to immune signaling in the cytoplasm? It seems that the activation of immunity can be triggered by different components of the DDR including DNA damage sensors, transducer kinases, and effectors.

**FIGURE 1:**
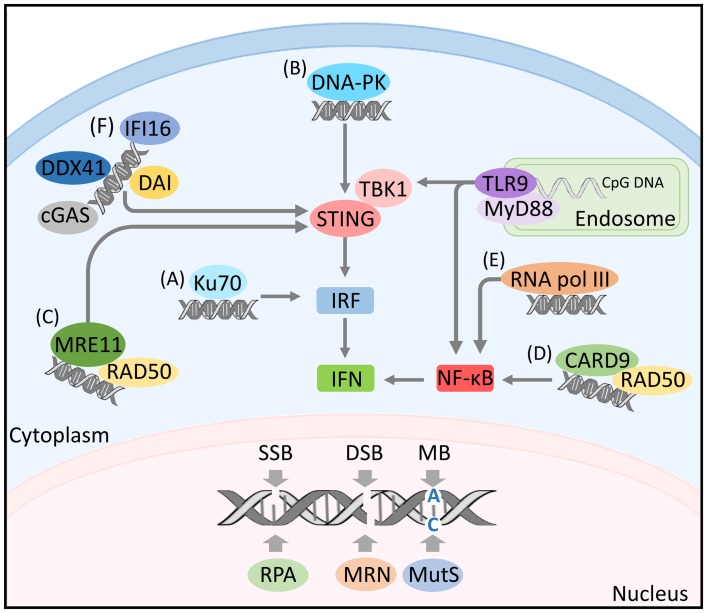
**DNA sensing and activation of immune signaling.** Nuclear DNA damage is recognized by a set of diverse sensors including: the protein complex of replication protein A (RPA) detecting single-strand breaks, the Mre11-Rad50-Nbs1 (MRN) complex sensing double-strand breaks and the MutS proteins recognizing mismatched bases. Single-stranded endosomal DNA activates TLR9 signaling. TLR9 recruits the myeloid differentiation marker 88 (MyD88) inducing the transcription of nuclear factor kappa B (NF-κB) and/or IFN-regulatory factor (IRF) by engaging with TANK-binding kinase 1 (TBK1). Cytosolic DNA can be detected by diverse DNA sensors including: **(A)** Ku70 activating IRF response. **(B)** DNA-PK mediating IRF response through engaging with TBK1/STING. **(C)** MRE-11 inducing IFN response by initiating STING-dependant signaling. **(D)** The innate immune adaptor CARD9 activating NF-κB. **(E)** RNA polymerase III inducing NF-κB activation. **(F)** The DNA-dependent activator of IFN regulatory factors (DAI), the interferon gamma-inducible protein 16 (IFI16), the DExD/H-box helicase 41 (DDX41) and the cGMP-AMP synthase (cGAS), all activating IFN response through TBK1/STING signaling.

DNA damage responses are initiated upon recognition of DNA lesions by specialized set of DNA sensors. These sensors specifically bind to diverse types of DNA lesions. Single-stranded DNA are recognized by a protein complex of replication protein A (RPA; Zou and Elledge, 2003), DSBs are detected by the Mre11-Rad50-Nbs1 (MRN) complex (Grenon et al., 2001), mismatched bases are recognized by MutS proteins (Pluciennik and Modrich, 2007), whereas damaged bases are sensed by DNA glycosylases (Lu et al., 1997; Hazra et al., 2003; **Figure 1**).

The role of DNA damage sensors in detecting aberrant DNA structures is not only restricted to the nucleus. Several studies showed that some DNA damage sensors play a role in the detection of foreign DNA in the cytoplasm leading to the activation of immune signaling. For instance, the DNA repair proteins, Ku70 and the DNA dependent protein kinase (DNA-PK), known to be involved in the detection of dsDNA breaks and the initiation of NHEJ (Ciccia and Elledge, 2010), act as pathogen recognition receptors (PRRs) for DNA in the cytoplasm. In response to cytosolic DNA, Ku70 induces the production of IFN-λ1 via the activation of IFN regulatory factor (IRF)-1 and IRF-7 (Zhang et al., 2011) (**Figure 1A**), while DNA-PK induces the expression of IFN-β, cytokine, and chemokine genes through the activation of IRF-3, TBK1, and STING (Ferguson et al., 2012) (**Figure 1B**). Additional evidence supporting a link between DNA damage and immunity revealed that the DNA damage sensor MRE11 recognizes cytosolic dsDNA but not viral DNA and initiates STING-dependent signaling leading to the induction of type I IFN (Kondo et al., 2013; **Figure 1C**). Moreover, the DNA-damage sensor Rad50 was shown to form a signaling complex with the innate immune adapter CARD9 in dendritic cells (DCs) upon their transfection with dsDNA or infection with viral DNA. The formation of dsDNA-Rad50-CARD9 signaling complexes induces NF-κB activation and pro-IL-1β generation (Roth et al., 2014; **Figure 1D**).

The Poly(ADP-ribose) polymerase-1 (PARP-1) has been implicated in multiple cellular processes such as DNA replication, transcription, DNA repair, apoptosis, and genome stability (reviewed in Bouchard et al. (2003)]. In addition, PARP-1 impacts the expression of NF-κB-dependent proinflammatory mediators such as TNFα, IL-6, and iNOS by inducing the translocation of NF-κB into the nucleus upon genotoxic stress (reviewed in Mangerich and Bürkle (2012)]. The enzyme called mutY Homolog (MUTYH) not only repairs oxidative DNA damage but has also been associated with circulating levels of IL-1 in healthy people (Sun et al., 2010) and levels of IL-1β and IL-6 in patients undergoing chronic hemodialysis (Cai et al., 2012). The 8-Oxoguanine DNA glycosylase-1 (OGG1) is a DNA glycosylase functioning in BER (Ba et al., 2014). OGG1-KO mice show a decrease in cytokine and chemokine production (Mabley et al., 2005), and a decrease in expression and translocation of STAT6 and NF-κB (Li et al., 2012). Another glycosylase involved in the BER-mediated removal of DNA lesions, the Apurinic/apyrimidinic endonuclease 1 (APE1), has been associated with the activation of immune signaling (Fung and Demple, 2005). APE-1 regulates transcription factors involved in inflammatory responses including NF-κB (Nishi et al., 2002), AP-1 (Xanthoudakis and Curran, 1992), HIF-1α (Huang et al., 1996, and p53 (Gaiddon et al., 1999). The transcriptional regulation of NF-κB and HIF-1α through APE-1 is thought to be necessary for the expression of TLR2-mediated inflammatory mediators, including TNF-α, CXCL8/IL-8, and LL-37, in human keratinocytes (Lee et al., 2009).

The DDR involves the PI3 kinase-like protein kinases ataxia telangiectasia mutated (ATM) and ataxia telangiectasia Rad-3 related (ATR). These kinases coordinate a DDR network when they are recruited to sites of DSBs and RPA (Shiloh, 2003). ATM and ATR signaling mediates DNA repair by inducing transcription of repair proteins and by recruiting repair factors to the site of DNA damage (Abraham, 2001). Within the context of DDR-mediated immune signaling, and after induction of DSBs, NF-kB essential modulator (NEMO), the regulatory subunit of IkB kinase (IKK) associates with ATM. This association activates IκB kinases and triggers NF-κB-dependent gene expression. Irradiated ATM knockout mice failed to induce canonical IKK activation compared to WT mice (Li et al., 2001).

The DDR does not only induce the production of proinflammatory signals such as IFNs, but also ligands that have the ability to bind to immune receptors. Examples of these receptors are the NKG2D, a member of the C-type lectin-like superfamily, and DNAX Accessory Molecule-1 (DNAM-1). NKG2D binds to the ligand MICA, MICB, ULBP1-6, a MHC class I-like protein. Whereas DNAM1 binds PVR/CD155 and Nectin-2/CD112 belonging to the Ig-like superfamily. These ligands are known to be induced by stress conditions such as cell divisions, viral infections and cancer reviewed in (Cerboni et al., 2015). NKG2D can activate NK cells, CD8+ T cells and γδ T cells (Champsaur and Lanier, 2010). Genotoxic stress and stalled DNA replication forks induced the expression of ligands for the NKG2D receptor in mouse and human cell lines (Gasser et al., 2005). Ligands’ upregulation required the activation of ATM or ATR protein kinases and DNA damage checkpoint pathways such as the Chk1 (a downstream transducer kinase in the pathway). Whereas exposure of cells to pharmacological or genetic inhibition of ATR, ATM or Chk1 prevented ligand upregulation. Moreover, siRNA knock-down of ATM in tumor cell lines abrogated NKG2D ligand expression (Gasser et al., 2005). Ligand expression of DNAM-1 was as well enhanced upon DDR. The treatment of multiple myeloma (MM) cells with low doses of chemotherapeutic drugs triggered the expression not only of NKG2D, but also of DNAM-1 ligands in an ATM/ATR-dependent manner promoting cellular adhesion to cells expressing DNAM-1 ligands including CD155 and CD112 (Bottino et al., 2003; Soriani et al., 2009). Taken all together, these findings demonstrate the ability of DNA damage sensors and DDR to activate immune signaling.

## Immune Stimulatory Effects of Nucleic Acid

Viral infections impose a challenging threat on human health. Unlike other microorganisms such as bacteria and fungi, viruses do not display microbe-specific patterns. Therefore, the repertoire of PRRs responsible for virus detection has evolved the ability to detect nucleic acids, a common pattern for all viruses. In vertebrates, foreign DNA can be recognized by two complementary nucleic acid detection systems: membrane bound PRRs (endosomal sensors) and cytoplasmic PRRs, both activating antiviral defense accounting essentially for type I interferon (IFNs) production (Stetson and Medzhitov, 2006).

So far TLR9 is the only known endosomal DNA sensor (**Figure 1**). This PRR is expressed in plasmacytoid DCs (Kadowaki et al., 2001) and responds to microbial oligodeoxynucleotides containing unmethylated CpG motifs (CpG-ODNs) derived from bacteria and viruses (Hemmi et al., 2000). The activation of TLR9 by its microbial ligand requires the internalization and endosomal maturation of CpG-DNA (Ahmad-Nejad et al., 2002). TLR9 activation is initiated by its transport and localization from the endoplasmic reticulum to the endolysosomes in dendritic cells where its ectodomains are cleaved (Ewald et al., 2008). Truncated TLR9 recruits the TLR adaptor myeloid differentiation marker 88 (MyD88) thus leading to the induction of inflammatory genes through the transcription of nuclear factor kappa B (NF-κB) and IFN-regulatory factor 7 (IRF-7). Details on TLR9 dependent induction of IFN regulatory factors is reviewed in (Takeuchi and Akira, 2010). The presence of DNA sensors and the restriction of their activity in recognizing nucleic acid and initiating signal transduction in intracellular compartments must have a protective effect against autoimmunity caused by recognition of self nucleic acids. TLR9 is one of the best examples in this respect as only the cleaved form of TLR9 recruits MyD88 (Ewald et al., 2008).

In contrast to endosomal sensing of DNA, cytosolic DNA sensing involves a diverse set of proteins acting as PRRs for nucleic acids. The recognition of cytosolic DNA by such sensors triggers signaling through TANK binding kinase 1 (TBK1) and its downstream transcription factor 3 (IRF3), leading to the production of type I IFN. Activation of TBK-1/IRF-3 signaling axis upon cytosolic DNA sensing is mediated by the stimulator of IFN genes (STING), a transmembrane protein acting as a signaling adaptor (Ishikawa et al., 2009).

The first discovered cytoplasmic sensor was the DNA-dependent activator of IFN regulatory factors (DAI), a Z-DNA binding protein named previously DLM-1. DAI’s expression was shown to be greatly up-regulated in the peritoneal lining tissue of tumor-bearing mice. The up-regulation of DAI was stimulated in macrophages by INF-γ or LPS suggesting that this protein plays a role in host defense (Fu et al., 1999). Later, it was shown that DAI binds to dsDNA enhancing its association with the IRF3 transcription factor and the TBK1 serine/threonine kinase and regulating the type I IFN response (Takaoka et al., 2007; **Figure 1F**). Another DNA sensor involved in innate immune responses is the RNA polymerase III. This polymerase recognizes AT rich dsRNA. By doing so, it activates RIG-1 and induces the production of type I interferon and the activation of the transcription factor NF-κB (Ablasser et al., 2009; **Figure 1E**). Inhibition of RNA pol III led to the abrogation of IFN-β induction upon infection with *Legionella pneumophila* and enhancement of bacterial growth (Chiu et al., 2009).

Cytosolic DNA can also be sensed by the interferon gamma-inducible protein 16 (IFI16). IR-induced DNA damage leads to nuclear localization of IFI16 and the formation of BRAC-1-IFI16 complex at genomic sites of DNA damage. This complex engages in the p53-mediated transmission of DNA damage signals and apoptosis (Aglipay et al., 2003). IFI16 functions as a DNA sensor in both the nucleus and the cytoplasm (Li et al., 2012) and as a nuclear pathogen sensor upon infection with Kaposi Sarcoma-associated herpesvirus (Kerur et al., 2011; **Figure 1F**). DExD/H-box helicase 41 (DDX41) also recognizes cytosolic DNA and DNA virus. DDX41 was found in the cytosol of myeloid dendritic cells (mDCs) together with STING. Upon its knockdown by shRNA mDCs failed to mount type I interferon and cytokine responses to DNA. Moreover, its KD blocked the activation of TBK1 and the transcription factors NF-κB and IRF3 (Zhang et al., 2011; **Figure 1F**). Another cytosolic DNA sensor is the cGMP-AMP synthase (cGAS). It was shown that cGAS has a second messenger function allowing its binding to STING, thus the induction of type I IFN response (Burdette et al., 2011; **Figure 1F**).

Taken together, the various cytosolic sensors play an integral role in the DNA-mediated activation of immune responses.

The detection of nucleic acids can be harmful for the host when it results in an excessive activation of immune cascades that can be costly in terms of energy, cause tissue damage and promote autoimmune diseases. Thus, cells have evolved in parallel fast and effective mechanisms for degrading DNA coming from pathogens, apoptotic cells or DNA replication byproducts. To rapidly degrade nucleic acids, cells utilize a set of DNases. Among these cellular DNases are DNases II that are present in macrophages. These enzymes degrade DNA of apoptotic cells engulfed by macrophages when the apoptotic enzyme caspase-activated DNase (CAD) failed to sufficiently digest chromosomal DNA (Kawane et al., 2003). Other DNases are the three prime repair exonuclease 1 (TREX1). These enzymes are found in the cytoplasm where they degrade DNA coming from endogenous retroviruses and DNA replication by-products. Cells deficient in TREX1 accumulate endogeneous single-stranded DNA (Yang et al., 2007; Stetson et al., 2008). Moreover, a loss of function mutation in the human Trex1 gene cause Aicardi–Goutieres syndrome (AGS), an autoimmune disorder (Crow et al., 2006).

## Chronic Inflammation as an Outcome of the DDR – Immune Signaling Cross Talk

An inflammation is a protective response mediating the elimination of injurious agents, the removal of necrotic cells and the initiation of tissue repair. Despite its beneficial effects, a prolonged inflammatory response can cause harm such as injuries in bystander normal tissues and promote inflammatory diseases. As a paradigm of the interplay between DDR and immunity, a prolonged or elevated inflammation can be an outcome of a persisting DDR or an accumulation of DNA damage due to deficiency in repair mechanisms. Mice carrying a Werner Syndrome (WS) mutation and a simultaneous knockdown of the RecQ-type DNA helicases exhibited an increased inflammatory status characterized by expression changes in HIF-1, IL-6, and components of the NFκB pathway (Turaga et al., 2009). Other studies showed that DDR can associate with autoimmune diseases. For instance, Schild-Poulter et al. (2008) identified in the serum of patients having systemic autoimmune rheumatic disease (SARD) autoantibodies against Ku, DNA-PKcs, poly (ADP-ribose) polymerase, and against DNA repair proteins such as Werner and Mre11. Additional studies reflecting the role of DNA damage in promoting autoimmune diseases showed that cell lines from patients with systemic lupus erythematosus (SLE) have a defective DSB repair (Davies et al., 2012). Moreover, low-density granulocytes (LDGs), an abnormal population of neutrophils found in SLE patients, have elevated levels of somatic alterations such as genetic damage compared to normal-density neutrophils (Singh et al., 2014). In the same context, Bawadekar et al. (2015) has demonstrated the presence of IFI16 in sera of systemic-autoimmune patients associated to an upregulation of cytokine encoding genes in endotoxin-free recombinant IFI16 (rIFI16) endothelial cells. IFI16 seemed to propagate inflammation in endothelial cells through the activation of p38 MAPK and NF-κB p65 (Bawadekar et al., 2015). Another example of DNA damage-related autoimmunity is provided by Karakasilioti et al. (2013) where they showed that persistent DNA damage signaling in mice, carrying a defective NER in adipose tissues only, triggers a chronic autoinflammatory response leading to fat depletion and metabolic abnormalities.

DNA damage driven inflammation can also promote tumourigenesis. For example, diethylnitrosamine (DEN)-induced hepatocellular carcinoma depends on IKKβ mediated inflammation. The carcinogen DEN causes DNA damage and leads to necrotic hepatocyte death resulting in the activation of inflammatory responses promoting tumor development (Maeda et al., 2005). In the same vein a defective DNA repair caused by a deficiency of the Fen1 exonuclease resulted in cancer initiation and a chronic inflammation promoting cancer progression (Zheng et al., 2007).

Persistent DDR triggers senescent cells to secrete growth factors, proteases, and inflammatory cytokines, termed the senescence-associated secretory phenotype (SASP; Freund et al., 2011). Cellular senescence is a tumor-suppressive mechanism arresting cells at risk for malignant transformation mediated by the tumor suppressor p53 upon DDR (Reinhardt and Schumacher, 2012). Despite the tumor-suppressing role associated with senescence, senescent cells can also induce deleterious changes in the tissue microenvironment promoting tumourigenesis (Coppe et al., 2010). Human cells bearing DSBs had an increase in secretion of inflammatory cytokines such as IL-6 and IL-8. Elevated cytokine secretion occurred upon persistent DDR and not upon transient DDR, suggesting that this increase in secretion is associated with senescence. Initiation and maintenance of cytokine signaling required the DDR proteins ATM, NBS1, and CHK2 but was independent of p53 (Rodier et al., 2009). In addition, the interleukin IL-1 signaling pathway was shown to be upregulated by senescent cells (Garfinkel and Brown, 1994). IL-1 can be secreted by senescent endothelial cells (Maier et al., 1990), fibroblasts (Palmieri et al., 1999), normal epithelial cells (Coppe et al., 2008) and epithelial cells in which senescence was induced by chemotherapy (Mantovani et al., 2001). SASP seems to promote cancer by cytokine-dependent growth of precancerous cells (Rodier et al., 2009).

## Chronic Inflammation as a Driving Force in the Genesis of DNA Damage and Malignancy

The majority of DDR studies have focused on physico-chemically induced aberrations in the genome. Only few studies have explored the role of *in vivo* physiological conditions, such as inflammation, in inducing DNA damage. Chronic inflammation has recently emerged as an important modulator of mutation susceptibility. Chronic inflammatory diseases such as colitis, hepatitis and pancreatitis induce oxidant-generating enzymes including NADPH oxidase and nitric oxide synthase (iNOS), thereby generating excessive production of mutagenic compounds such as ROS and reactive nitrogen species (RNS; Bartsch and Nair, 2006). ROS/RNS produced by neutrophils and macrophages (Coussens and Werb, 2002) can cause damage to nuclear and mitochondrial DNA (Wiseman and Halliwell, 1996). Damage is induced by nitration, oxidation, methylation and deamination reactions causing alterations in the DNA structure that can ultimately lead to mutations, rearrangements, deletions and insertions and indirectly lead to base alkylation via lipid peroxidation (LPO; Wiseman and Halliwell, 1996). For example, an increase in oxidative base damage has been observed in the case of some chronic inflammatory diseases such as hepatitis (Hagen et al., 1994) and rheumatoid arthritis (Bashir et al., 1993). In parallel to causing DNA damage, ROS/RNS can cause oxidative protein damage modifying the activity of DNA polymerases, thereby impairing DNA repair pathways (Wink et al., 1998). ROS/RNS can as well modify the function of proteins involved in cell proliferation and differentiation (Wiseman and Halliwell, 1996). Accumulation of DNA damage caused by either an increase in oxidative damage or a decrease in repair efficiency, leads to malignant diseases. For instance, oxidative DNA damage occurring in tumor-suppressor genes, oncogenes and key regulators of cell proliferation can promote tumourigenesis (Meira et al., 2008). Cells cultured under oxidative stress conditions develop malignant transformation (Zimmerman and Cerutti, 1984; Weitzman and Gordon, 1990) and a defective MMR causing hereditary non-polyposis colon cancer (Marx, 1994). Taken together, chronic inflammation accompanied by the generation of ROS/RNS drives the transformation of normal cells into malignant cells through the production of oxidative DNA damage and the impairment of DNA repair pathways.

Reactive oxygen species/RNS-derived DNA lesions are repaired by BER upon their recognition by specific glycosylases. A link between glycosylases deficient in removing ROS/RNS-derived base lesions and cancer development has been established. For example, mice carrying mutations in the MYH glycosylase that removes 8-Oxoguanine (8oxoG), a DNA lesion resulting from ROSs, were reported more susceptible to oxidative-induced intestinal tumors (Sakamoto et al., 2007). Moreover, mice deficient in 8-Oxoguanine DNA glycosylase 1 (Ogg1) were found to be more susceptible to lung cancer (Sakumi et al., 2003). Wild-type alkyladenine DNA glycosylase (Aag) mice with inflammatory bowel diseases were protected against colonic epithelial damage and colon tumourigenesis through an Aag-mediated DNA repair pathway, whereas Aag-deficient mice with the same colonic inflammation as WT animals had a high accumulation of ROS/RNS-derived DNA base lesions followed by severe gastric lesions and colon carcinogenesis (Meira et al., 2008).

There is growing evidence demonstrating that chronic inflammation induces ROS/RNS-derived DNA damage promoting several human cancers. For instance, liver cancer was related to infection with chronic viral hepatitis B. HBV-infected patients with chronic hepatitis and liver cirrhosis had a massive increase in DNA repair markers in their urine compared to asymptomatic HBV-carriers. The induction of repair of damaged DNA could be related to HBV-induced chronic inflammation causing DNA lesions through an excessive production of ROS and RNS (Nair et al., 2002). Gastric cancer was shown as well to be associated with chronic inflammation induced by infection with *Helicobacter pylori* (Correa, 1994). This could be explained by the observed increase in iNOS expression and oxidative damage in gastric mucosa cells with *H. pylori* infection (Iacopini et al., 2003). Chronic intestinal inflammation manifested by Crohn’s disease (CD) can promote colorectal cancer. Levels of ROS/RNS were reported to be elevated in colonic mucosa of CD patients and correlated with disease severity (Bartsch and Nair, 2006). Additionally, chronic bladder inflammation upon infection with *Schistosoma haematobium* has been associated with increased cancer. Patients with bladder cancer showed genetic alterations in chromosome 11 and insertion of a normal chromosome 11 promoted protection of cells against bladder carcinoma (Rosin et al., 1994). Consistently, an increase in ROS-mediated DNA damage has been observed in tissues with ductal carcinoma compared to control tissues (Malins and Haimanot, 1991) and in cases of breast inflammatory diseases (Jaiyesimi and Buzdar, 1992). Another example revealed that cholangiocarcinoma, a cancer arising from cells within the bile ducts was shown to be associated with inflammatory responses. Exposure of human cholangiocarcinoma cell lines to inflammatory cytokines such as IL-1β, IFN-γ, and TNF-α led to the activation of iNOS and the excessive production of nitric oxide (NO) which caused DNA damage and impairment of DNA repair (Jaiswal et al., 2000). Moreover, exposure to asbestos may cause asbestosis, which is an inflammatory condition affecting lungs and causing shortness of breath and coughing. Some asbestos types, such as crocidolite induce release of ROS from neutrophils and macrophages, increasing then level of oxidative DNA damage in human promyelocytic leukemia cell line (HL60; Takeuchi and Morimoto, 1994). Inflammation has been shown as well to act synergistically with DNA damage in order to induce mutations driving cancer development. Induction of DSBs (as assessed by γH2AX foci) and an increase in cell proliferation were observed in mice exposed to cerulein, a potent inducer of pancreatic inflammation. Both, inflammation-induced DNA damage and inflammation-induced cell proliferation significantly induced HR (Kiraly et al., 2015).

Nonetheless, chronic inflammation can cause DNA damage independently from the release of ROS/RNS. Loss of protective mucus occurring upon chronic inflammation increases intestinal permeability for toxins and mutagens inducing mutations and promoting cancer (Sakaguchi and Brand, 2001). The connection between chronic inflammation and tumourigenesis is further supported by findings demonstrating that inflammatory mediators cause genetic instability by leading to accumulation of random genetic alterations in cancer cells. Examples of key players of cancer-related inflammation (CRI) include tumor-infiltrating lymphocytes, tumour-associated macrophages (TAMs), the secretion of cytokines such as TNF, IL-1, IL-6, and chemokines such as CCL2 and CXCL8, in addition to the occurrence of tissue remodeling and angiogenesis (Colotta et al., 2009). The secretion of cytokines activates the oncogenic transcription factor NF-κB and STAT3, both inducing the expression of target genes crucial for tumourigenesis such as anti-apoptotic genes, stress-response genes and pro-angiogenic molecules reviewed in (Grivennikov and Karin, 2010). A recent study showed that chronic inflammation induced by knockout of the nfkb1 subunit of NF-κB in mice caused premature aging, reduced regeneration in liver and gut, and stabilized DNA damage via ROS-mediated exacerbation of telomere dysfunction and cell senescence (Jurk et al., 2014).

**FIGURE 2:**
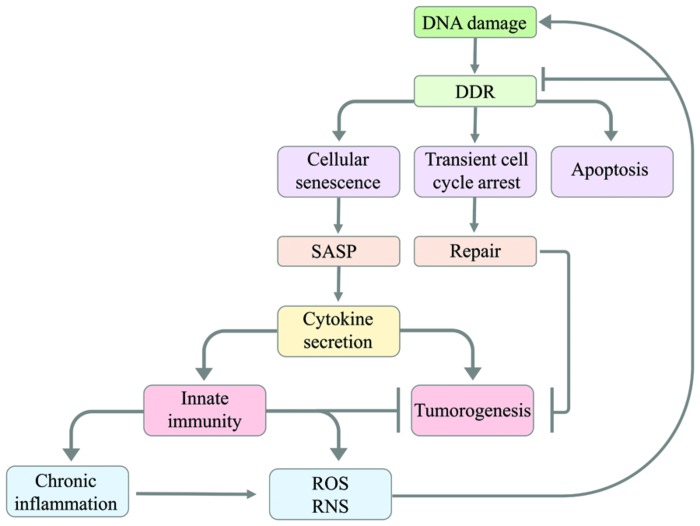
**Model illustrating how DNA damage leads to the activation of innate immunity and how innate immunity causes in return DNA damage.** The DNA damage response leads to apoptosis, transient cell cycle arrest or cellular senescence. Transient cell cycle arrest has a protective effect against tumourigenesis as it allows cells to accurately repair DNA damage before cell cycle progression. Cellular senescence can cause senescent cells to modify their tissue environment through the senescence-associated secretory phenotype (SASP) resulting in cytokine secretion that activates the innate immune system. The innate immune system can suppress tumourigenesis by clearing senescent cells with oncogene activation or chronic DNA damage. However, SASP can also cause tumourigenesis through cytokine signaling promoting the proliferation of tumor cells. The activation of innate immunity involves the production of reactive oxygen species (ROS) and reactive nitrogen species (RNS) and can promote chronic inflammation. The generation of ROS/RNS by innate immunity and chronic inflammation promotes tumourigenesis through causing mutations in neighboring cells, thus triggering DNA damage, or impairing DDR.

In summary, chronic inflammatory responses caused by infection, autoimmune diseases or exposure to irritants in selected organs promote cancer through an increase in DNA damage and inhibition of DNA repair pathways (**Figure 2**). Yet deeper insight into the intricate connections between inflammatory responses and tumourigenesis is required for developing efficient therapies aiming at suppressing pro-tumourigenic pathways and enhancing anti-tumor immunity.

## Concluding Remarks

The interplay between DDR and immune signaling has important implications for the organisms’ response to genome aberrations. A tight functional connection and coordination between DDR and immune defense promotes protection from infectious microorganisms and surveillance against tumors. Considerable progress has been made in deepening our knowledge on the role of DDR in the function of the immune system. Recent experimental data demonstrated a role of DNA damage sensors in inducing inflammatory responses, triggering processes linked to host defense against microbial infection, and in promoting autoimmune diseases upon detection of both nuclear and cytosolic DNA products.

Further studies are required to improve our understanding on the interaction or redundancy between the diverse proposed DNA sensors and on how the DNA sensing machinery discriminates foreign from self-damaged DNA. Deeper insight into this machinery might also impact the development of DNA virus-based vaccine vectors. Moreover, the mediators linking the activation of immunity in the cytosol upon DNA damage recognition in the nucleus await their investigation. The characterization of these mediators should aid finding therapeutic strategies that facilitate the recognition of cells with genomic aberrations such as cancer cells. Finally, research in this area is likely to reveal new therapeutic interventions against diseases such as cancer, age-related pathologies, autoimmune and chronic inflammatory diseases.

## Author Contributions

RN and BS wrote the manuscript and made substantial, direct and intellectual contribution to the work, and approved it for publication.

## Conflict of Interest Statement

The authors declare that the research was conducted in the absence of any commercial or financial relationships that could be construed as a potential conflict of interest.

## References

[B1] Ablasser A. Bauernfeind F. Hartmann G. Latz E. (2009). RIG-I-dependent sensing of poly (dA: dT) through the induction of an RNA polymerase III–transcribed RNA intermediate. *Nat. Immunol.* 10 1065–1072. 10.1038/ni.1779 19609254 PMC3878616

[B2] Abraham R. T. (2001). Cell cycle checkpoint signalling through the ATM and ATR kinases. *Genes Dev.* 15 2177–2196. 10.1101/gad.914401 11544175

[B3] Aglipay J. A. Lee S. W. Okada S. Fujiuchi N. Ohtsuka T. (2003). A member of the Pyrin family, IFI 16, is a novel BRCA1-associated protein involved in the p53-mediated apoptosis pathway. *Oncogene* 22 8931–8938. 10.1038/sj.onc.1207057 14654789

[B4] Ahmad-Nejad P. Häcker H. Rutz M. (2002). Bacterial CpG-DNA and lipopolysaccharides activate Toll-like receptors at distinct cellular compartments. *Eur. J. Immunol.* 32 1958–1968. 12115616 10.1002/1521-4141(200207)32:7<1958::AID-IMMU1958>3.0.CO;2-U

[B5] Ank N. West H. Bartholdy C. Eriksson K. (2006). Lambda interferon (IFN-λ), a type III IFN, is induced by viruses and IFNs and displays potent antiviral activity against select virus infections in vivo. *J. Virol.* 80 4501–4509. 10.1128/JVI.80.9.4501-4509.2006 16611910 PMC1472004

[B6] Ba X. Bacsi A. Luo J. Aguilera L. (2014). 8-oxoguanine DNA glycosylase-1 augments proinflammatory gene expression by facilitating the recruitment of site-specific transcription factors. *J. Immunol.* 192 2384–2394. 10.4049/jimmunol.1302472 24489103 PMC3943862

[B7] Baldwin E. L. Osheroff N. (2005). Etoposide, topoisomerase II and cancer. *Curr. Med. Chem. Anti Cancer Agents* 5 363–372. 16101488 10.2174/1568011054222364

[B8] Bartsch H. Nair J. (2006). Chronic inflammation and oxidative stress in the genesis and perpetuation of cancer: role of lipid peroxidation, DNA damage, and repair. *Langenbecks Arch. Surg.* 391 499–510. 10.1007/s00423-006-0073-1 16909291

[B9] Bashir S. Harris G. Denman M. A. Blake D. R. (1993). Oxidative DNA damage and cellular sensitivity to oxidative stress in human autoimmune diseases. *Ann. Rheum. Dis.* 52 659–666. 10.1136/ard.52.9.659 8239761 PMC1005143

[B10] Bauer N. C. Corbett A. H. Doetsch P. W. (2015). The current state of eukaryotic DNA base damage and repair. *Nucleic Acids Res.* 43 10083–10101. 10.1093/nar/gkv1136 26519467 PMC4666366

[B11] Bawadekar M. De Andrea M. Lo Cigno I. Baldanzi G. Caneparo V. Graziani A. (2015). The extracellular IFI16 protein propagates inflammation in endothelial cells via p38 MAPK and NF-κB p65 activation. *J. Interferon Cytokine Res.* 35 441–453. 10.1089/jir.2014.0168 25715050 PMC4490711

[B12] Bottino C. Castriconi R. Pende D. Rivera P. (2003). Identification of PVR (CD155) and Nectin-2 (CD112) as cell surface ligands for the human DNAM-1 (CD226) activating molecule. *J. Exp. Med.* 198 557–567. 10.1084/jem.20030788 12913096 PMC2194180

[B13] Bouchard V. J. Rouleau M. Poirier G. G. (2003). PARP-1, a determinant of cell survival in response to DNA damage. *Exp. Hematol.* 31 446–454. 10.1016/S0301-472X(03)00083-3 12829019

[B14] Brzostek S. (2011). The DNA damage response induces IFN. *J. Immunol.* 187 5336–5345. 10.4049/jimmunol.1100040 22013119 PMC3246365

[B15] Burdette D. L. Monroe K. M. Sotelo K. (2011). STING is a direct innate immune sensor of cyclic di-GMP. *Nature* 478 515–518. 10.1038/nature10429 21947006 PMC3203314

[B16] Cai Z. Guo W. Chen H. Tao J. Cao L. Sun W. (2012). Base excision repair gene polymorphisms are associated with inflammation in patients undergoing chronic hemodialysis. *Biochem. Biophys. Res. Commun.* 424 611–615. 10.1016/j.bbrc.2012.06.161 22780951

[B17] Caldecott K. W. (2008). Single-strand break repair and genetic disease. *Nat. Rev. Genet.* 9 619–631. 10.1038/nrg2380 18626472

[B18] Cerboni C. Fionda C. Soriani A. Zingoni A. (2015). The DNA damage response: a common pathway in the regulation of NKG2D and DNAM-1 ligand expression in normal, infected, and cancer cells. *Front. Immunol.* 4:508 10.3389/fimmu.2013.00508 PMC3882864 24432022

[B19] Champsaur M. Lanier L. L. (2010). Effect of NKG2D ligand expression on host immune responses. *Immunol. Rev.* 235 267–285. 10.1111/j.0105-2896.2010.00893.x 20536569 PMC2885032

[B20] Chiu Y. H. MacMillan J. B. Chen Z. J. (2009). RNA polymerase III detects cytosolic DNA and induces type I interferons through the RIG-I pathway. *Cell* 138 576–591. 10.1016/j.cell.2009.06.015 19631370 PMC2747301

[B21] Ciccia A. Elledge S. J. (2010). The DNA damage response: making it safe to play with knives. *Mol. Cell* 40 179–204. 10.1016/j.molcel.2010.09.019 20965415 PMC2988877

[B22] Colotta F. Allavena P. Sica A. Garlanda C. (2009). Cancer-related inflammation, the seventh hallmark of cancer: links to genetic instability. *Carcinogenesis* 30 1073–1081. 10.1093/carcin/bgp127 19468060

[B23] Coppe J. P. Desprez P. Y. Krtolica A. (2010). The senescence-associated secretory phenotype: the dark side of tumour suppression. *Annu. Rev. Pathol.* 5 99–118. 10.1146/annurev-pathol-121808-102144 20078217 PMC4166495

[B24] Coppe J. P. Patil C. K. Rodier F. Sun Y. Munoz D. P. (2008). Senescence-associated secretory phenotypes reveal cell-nonautonomous functions of oncogenic RAS and the p53 tumour suppressor. *PLoS Biol.* 6:e301 10.1371/journal.pbio.0060301 PMC2592359 19053174

[B25] Correa P. (1994). *Helicobacter pylori* and gastric carcinogenesis. *Am. J. Surg. Pathol.* 19 S37–S43. 7762738

[B26] Coussens L. M. Werb Z. (2002). Inflammation and cancer. *Nature* 420 860–867. 10.1038/nature01322 12490959 PMC2803035

[B27] Crow Y. J. Hayward B. E. Parmar R. Robins P. Leitch A. (2006). Mutations in the gene encoding the 3’-5’ DNA exonuclease TREX1 cause Aicardi-Goutieres syndrome at the AGS1 locus. *Nat. Genet.* 38 917–920. 10.1038/ng1845 16845398

[B28] Davies R. C. Pettijohn K. Fike F. Wang J. (2012). Defective DNA double-strand break repair in pediatric systemic lupus erythematosus. *Arthritis Rheum.* 64 568–578. 10.1002/art.33334 21905016

[B29] Edifizi D. Schumacher B. (2015). Genome instability in development and aging: insights from nucleotide excision repair in humans, mice, and worms. *Biomolecules* 5 1855–1869. 10.3390/biom5031855 26287260 PMC4598778

[B30] Ermolaeva M. A. Schumacher B. (2014a). Insights from the worm: the *C. elegans* model for innate immunity. *Semin. Immunol.* 26 303–309. 10.1016/j.smim.2014.04.005 24856329 PMC4248339

[B31] Ermolaeva M. A. Schumacher B. (2014b). Systemic DNA damage responses: organismal adaptations to genome instability. *Trends Genet.* 30 95–102. 10.1016/j.tig.2013.12.001 24439457 PMC4248340

[B32] Ermolaeva M. A. Segref A. Dakhovnik A. Ou H. L. Schneider J. I. Utermöhlen O. (2013). DNA damage in germ cells induces an innate immune response that triggers systemic stress resistance. *Nature* 501 416–420. 10.1038/nature12452 23975097 PMC4120807

[B33] Ewald S. E. Lee B. L. Lau L. Wickliffe K. E. Shi G. P. (2008). The ectodomain of Toll-like receptor 9 is cleaved to generate a functional receptor. *Nature* 456 658–662. 10.1038/nature07405 18820679 PMC2596276

[B34] Ferguson B. J. Mansur D. S. Peters N. E. Ren H. Smith G. L. (2012). DNA-PK is a DNA sensor for IRF-3-dependent innate immunity. *Elife* 1:e00047 10.7554/eLife.00047.001 PMC3524801 23251783

[B35] Freund A. Patil C. K. Campisi J. (2011). p38MAPK is a novel DNA damage response-independent regulator of the senescence-associated secretory phenotype. *EMBO J.* 30 1536–1548. 10.1038/emboj.2011.69 21399611 PMC3102277

[B36] Fu Y. Comella N. Tognazzi K. Brown L. F. Dvorak H. F. (1999). Cloning of DLM-1, a novel gene that is up-regulated in activated macrophages, using RNA differential display. *Gene* 240 157–163. 10.1016/S0378-1119(99)00419-9 10564822

[B37] Fung H. Demple B. (2005). A vital role for Ape1/Ref1 protein in repairing spontaneous DNA damage in human cells. *Mol. Cell* 17 463–470. 10.1016/j.molcel.2004.12.029 15694346

[B38] Gaiddon C. Moorthy N. C. Prives C. (1999). Ref-1 regulates the transactivation and pro-apoptotic functions of p53 in vivo. *EMBO J.* 18 5609–5621. 10.1093/emboj/18.20.5609 10523305 PMC1171629

[B39] Garfinkel S. Brown S. (1994). Post-transcriptional regulation of interleukin 1 alpha in various strains of young and senescent human umbilical vein endothelial cells. *Proc. Natl. Acad. Sci. U.S.A.* 91 1559–1563. 10.1073/pnas.91.4.1559 8108444 PMC43199

[B40] Gasser S. Orsulic S. Brown E. J. Raulet D. H. (2005). The DNA damage pathway regulates innate immune system ligands of the NKG2D receptor. *Nature* 436 1186–1190. 10.1038/nature03884 15995699 PMC1352168

[B41] Grenon M. Gilbert C. Lowndes N. F. (2001). Checkpoint activation in response to double-strand breaks requires the Mre11/Rad50/Xrs2 complex. *Nat. Cell Biol.* 3 844–847. 10.1038/ncb0901-844 11533665

[B42] Grivennikov S. I. Karin M. (2010). Dangerous liaisons: STAT3 and NF-κB collaboration and crosstalk in cancer. *Cytokine Growth Factor Rev.* 21 11–19. 10.1016/j.cytogfr.2009.11.005 20018552 PMC2834864

[B43] Hagen T. M. Huang S. Curnutte J. (1994). Extensive oxidative DNA damage in hepatocytes of transgenic mice with chronic active hepatitis destined to develop hepatocellular carcinoma. *Proc. Natl. Acad. Sci. U.S.A.* 91 12808–12812. 7809125 10.1073/pnas.91.26.12808 PMC45529

[B44] Halazonetis T. D. Gorgoulis V. G. Bartek J. (2008). An oncogene-induced DNA damage model for cancer development. *Science* 319 1352–1355. 10.1126/science.1140735 18323444

[B45] Hazra T. K. Izumi T. Kow Y. W. Mitra S. (2003). The discovery of a new family of mammalian enzymes for repair of oxidatively damaged DNA, and its physiological implications. *Carcinogenesis* 24 155–157. 10.1093/carcin/24.2.155 12584162

[B46] Hemmi H. Takeuchi O. Kawai T. Kaisho T. Sato S. (2000). A Toll-like receptor recognizes bacterial DNA. *Nature* 408 740–745. 10.1038/35047123 11130078

[B47] Hoeijmakers J. (2009). DNA damage, aging, and cancer. *N. Engl. J. Med.* 361 1475–1485. 10.1056/NEJMra0804615 19812404

[B48] Huang L. E. Arany Z. Livingston D. M. Bunn H. F. (1996). Activation of hypoxia-inducible transcription factor depends primarily upon redox-sensitive stabilization of its α subunit. *J. Biol. Chem.* 271 32253–32259. 10.1074/jbc.271.50.32253 8943284

[B49] Iacopini F. Consolazio A. Bosco D. (2003). Oxidative damage of the gastric mucosa in *Helicobacter pylori* positive chronic atrophic and nonatrophic gastritis, before and after eradication. *Helicobacter* 8 503–512. 10.1046/j.1523-5378.2003.00172.x 14535997

[B50] Isaacs A. Cox R. A. Rotem Z. (1963). Foreign nucleic acids as the stimulus to make interferon. *Lancet* 282 113–116. 10.1016/S0140-6736(63)92585-6 13956740

[B51] Ishikawa H. Ma Z. Barber G. N. (2009). STING regulates intracellular DNA-mediated, type I interferon-dependent innate immunity. *Nature* 461 788–792. 10.1038/nature08476 19776740 PMC4664154

[B52] Jackson S. P. Bartek J. (2009). The DNA-damage response in human biology and disease. *Nature* 461 1071–1078. 10.1038/nature08467 19847258 PMC2906700

[B53] Jaiswal M. LaRusso N. F. Burgart L. J. Gores G. J. (2000). Inflammatory cytokines induce DNA damage and inhibit DNA repair in cholangiocarcinoma cells by a nitric oxide-dependent mechanism. *Cancer Res.* 60 184–190. 10646872

[B54] Jaiyesimi I. A. Buzdar A. U. (1992). Inflammatory breast cancer: a review. *J. Clin. Oncol.* 10 1014–1024. 1588366 10.1200/JCO.1992.10.6.1014

[B55] Jiricny J. (2006). The multifaceted mismatch-repair system. *Nat. Rev. Mol. Cell Biol.* 7 335–346. 10.1038/nrm1907 16612326

[B56] Jurk D. Wilson C. Passos J. F. Oakley F. (2014). Chronic inflammation induces telomere dysfunction and accelerates ageing in mice. *Nat. Commun.* 2 4172 10.1038/ncomms5172 PMC4090717 24960204

[B57] Kadowaki N. Ho S. Antonenko S. (2001). Subsets of human dendritic cell precursors express different toll-like receptors and respond to different microbial antigens. *J. Exp. Med.* 194 863–870. 10.1084/jem.194.6.863 11561001 PMC2195968

[B58] Karakasilioti I. Kamileri I. Chatzinikolaou G. Kosteas T. (2013). DNA damage triggers a chronic autoinflammatory response, leading to fat depletion in NER progeria. *Cell Metab.* 18 403–415. 10.1016/j.cmet.2013.08.011 24011075 PMC4116679

[B59] Kawane K. Fukuyama H. Yoshida H. Nagase H. (2003). Impaired thymic development in mouse embryos deficient in apoptotic DNA degradation. *Nat. Immunol.* 4 138–144. 10.1038/ni881 12524536

[B60] Kerur N. Veettil M. V. Sharma N. (2011). IFI16 acts as a nuclear pathogen sensor to induce the inflammasome in response to Kaposi Sarcoma-associated herpesvirus infection. *Cell Host Microbe* 9 363–375. 10.1016/j.chom.2011.04.008 21575908 PMC3113467

[B61] Kiraly O. Gong G. Olipitz W. Muthupalani S. (2015). Inflammation-induced cell proliferation potentiates DNA damage-induced mutations in vivo. *PLoS Genet.* 11:e1004901 10.1371/journal.pgen.1004901 PMC4372043 25647331

[B62] Kondo T. Kobayashi J. Saitoh T. (2013). DNA damage sensor MRE11 recognizes cytosolic double-stranded DNA and induces type I interferon by regulating STING trafficking. *Proc. Natl. Acad. Sci. U.S.A.* 110 2969–2974. 10.1073/pnas.1222694110 23388631 PMC3581880

[B63] Krieg A. M. (2002). CPG motifs in bacterial DNA and their immune effects. *Annu. Rev. Immunol.* 20 709–760. 10.1146/annurev.immunol.20.100301.064842 11861616

[B64] Lee H. M. Yuk J. M. Shin D. M. Yang C. S. (2009). Apurinic/apyrimidinic endonuclease 1 is a key modulator of keratinocyte inflammatory responses. *J. Immunol.* 183 6839–6848. 10.4049/jimmunol.0901856 19846872

[B65] Li G. Yuan K. Yan C. Fox J. Gaid M. (2012). 8-Oxoguanine-DNA glycosylase 1 deficiency modifies allergic airway inflammation by regulating STAT6 and IL-4 in cells and in mice. *Free Radic. Biol. Med.* 52 392–401. 10.1016/j.freeradbiomed.2011.10.490 22100973 PMC3740570

[B66] Li N. Banin S. Ouyang H. Li G. C. Courtois G. Shiloh Y. (2001). ATM is required for IkappaB kinase (IKKk) activation in response to DNA double strand breaks. *J. Biol. Chem.* 276 8898–8903. 10.1074/jbc.M009809200 11114307

[B67] Li T. Diner B. A. Chen J. (2012). Acetylation modulates cellular distribution and DNA sensing ability of interferon-inducible protein IFI16. *Proc. Natl. Acad. Sci. U.S.A.* 109 10558–10563. 10.1073/pnas.1203447109 22691496 PMC3387042

[B68] Lindahl T. Barnes D. E. (2000). Repair of endogenous DNA damage. *Cold Spring Harb. Symp. Quant. Biol.* 65 127–134. 10.1101/sqb.2000.65.127 12760027

[B69] Lu R. Nash H. M. Verdine G. L. (1997). A mammalian DNA repair enzyme that excises oxidatively damaged guanines maps to a locus frequently lost in lung cancer. *Curr. Biol.* 7 397–407. 10.1016/S0960-9822(06)00187-4 9197244

[B70] Mabley J. G. Pacher P. Deb A. Wallace R. Elder R. H. (2005). Potential role for 8-oxoguanine DNA glycosylase in regulating inflammation. *FASEB J.* 19 290–292. 10.1096/fj.04-2278fje 15677345

[B71] Maeda S. Kamata H. Luo J. L. Leffert H. Karin M. (2005). IKKβ couples hepatocyte death to cytokine-driven compensatory proliferation that promotes chemical hepatocarcinogenesis. *Cell* 121 977–990. 10.1016/j.cell.2005.04.014 15989949

[B72] Maier J. A. Voulalas P. Roeder D. Maciag T. (1990). Extension of the life-span of human endothelial cells by an interleukin-1 alpha antisense oligomer. *Science* 249 1570–1574. 10.1126/science.2218499 2218499

[B73] Malins D. C. Haimanot R. (1991). Major alterations in the nucleotide structure of DNA in cancer of the female breast. *Cancer Res.* 51 5430–5432. 1655250

[B74] Mangerich A. Bürkle A. (2012). Pleiotropic cellular functions of PARP1 in longevity and aging: genome maintenance meets inflammation. *Oxid. Med. Cell. Longev.* 2012:321653 10.1155/2012/321653 PMC3459245 23050038

[B75] Mantovani A. Locati M. Vecchi A. Sozzani S. (2001). Decoy receptors: a strategy to regulate inflammatory cytokines and chemokines. *Trends Immunol.* 22 328–336. 10.1016/S1471-4906(01)01941-X 11377293

[B76] Marx J. (1994). DNA repair comes into its own. *Science* 266 728–730. 10.1126/science.7973626 7973626

[B77] Meira L. B. Bugni J. M. Green S. L. Lee C. W. (2008). DNA damage induced by chronic inflammation contributes to colon carcinogenesis in mice. *J. Clin. Invest.* 118 2516–2525. 10.1172/JCI35073 18521188 PMC2423313

[B78] Mendoza J. Martínez J. Hernández C. Pérez-Montiel D. Castro C. Fabián-Morales E. (2013). Association between ERCC1 and XPA expression and polymorphisms and the response to cisplatin in testicular germ cell tumours. *Br. J. Cancer* 109 68–75. 10.1038/bjc.2013.303 23807173 PMC3708571

[B79] Meresse P. Dechaux E. Monneret C. Bertounesque E. (2004). Etoposide: discovery and medicinal chemistry. *Curr. Med. Chem.* 11 2443–2466. 15379707 10.2174/0929867043364531

[B80] Nair J. Srivatanakul P. Jedpiyawongse A. Bartsch H. (2002). Urinary excretion of 1, N6-ethenodeoxyadenosine in patients diagnosed with chronic hepatitis, liver cirrhosis and hepatocellular carcinoma from Thailand. *Proc. AACR* 42:2843.

[B81] Nishi T. Shimizu N. Hiramoto M. Sato I. (2002). Spatial redox regulation of a critical cysteine residue of NF-κB in vivo. *J. Biol. Chem.* 277 44548–44556. 10.3410/f.1002390.167758 12213807

[B82] Palmieri D. Watson J. M. Rinehart C. A. (1999). Age-related expression of PEDF/EPC-1 in human endometrial stromal fibroblasts: implications for interactive senescence. *Exp. Cell Res.* 247 142–147. 10.1006/excr.1998.4341 10047456

[B83] Pan M. R. Li K. Lin S. Y. Hung W. C. (2016). Connecting the dots: from DNA damage and repair to aging. *Int. J. Mol. Sci.* 17:685 10.3390/ijms17050685 PMC4881511 27164092

[B84] Pateras I. S. Havaki S. Nikitopoulou X. Vougas K. Townsend P. A. Panayiotidis M. I. (2015). The DNA damage response and immune signalling alliance: is it good or bad? Nature decides when and where. *Pharmacol. Ther.* 154 36–56. 10.1016/j.pharmthera.2015.06.011 26145166

[B85] Pluciennik A. Modrich P. (2007). Protein roadblocks and helix discontinuities are barriers to the initiation of mismatch repair. *Proc. Natl. Acad. Sci. U.S.A.* 104 12709–12713. 10.3410/f.1088532.541604 17620611 PMC1913546

[B86] Reinhardt H. C. Schumacher B. (2012). The p53 network: cellular and systemic DNA damage responses in aging and cancer. *Trends Genet.* 28 128–136. 10.1016/j.tig.2011.12.002 22265392 PMC4120491

[B87] Ribezzo F. Shiloh Y. Schumacher B. (2016). Systemic DNA damage responses in aging and diseases. *Semin. Cancer Biol.* 37–38, 26–35. 10.1016/j.semcancer.2015.12.005 PMC4886830 26773346

[B88] Rodier F. Coppé J.-P. Patil C. K. Hoeijmakers W. A. M. Muñoz D. P. Raza S. R. (2009). Persistent DNA damage signalling triggers senescence-associated inflammatory cytokine secretion. *Nat. Cell Biol.* 11 973–979. 10.1038/ncb1909 19597488 PMC2743561

[B89] Rodriguez H. (2011). DNA damage and autophagy. *Mutat. Res.* 711 158–166. 10.1016/j.mrfmmm.2011.03.007 21419786 PMC3105359

[B90] Rosin M. P. Anwar W. A. Ward A. J. (1994). Inflammation, chromosomal instability, and cancer: the schistosomiasis model. *Cancer Res.* 54(7 Supplement), 1929s–1933s. 8137314

[B91] Roth S. Rottach A. Lotz A. S. (2014). Rad50-CARD9 interactions link cytosolic DNA sensing to IL-1β production. *Nat. Immunol.* 15 538–545. 10.1038/ni.2888 24777530 PMC4309842

[B92] Sakaguchi T. Brand S. (2001). Mucosal barrier and immune mediators. *Curr. Opin. Gastroenterol.* 17 573–577. 17031221 10.1097/00001574-200111000-00016

[B93] Sakamoto K. Tominaga Y. Yamauchi K. Nakatsu Y. (2007). MUTYH-null mice are susceptible to spontaneous and oxidative stress–induced intestinal tumourigenesis. *Cancer Res.* 67 6599–6604. 10.1158/0008-5472.CAN-06-4802 17638869

[B94] Sakumi K. Tominaga Y. Furuichi M. Xu P. Tsuzuki T. (2003). Ogg1 knockout-associated lung tumourigenesis and its suppression by Mth1 gene disruption. *Cancer Res.* 63 902–905. 12615700

[B95] Schild-Poulter C. Su A. Shih A. Kelly O. P. Fritzler M. J. Goldstein R. (2008). Association of autoantibodies with Ku and DNA repair proteins in connective tissue diseases. *Rheumatology* 47 165–171. 10.1093/rheumatology/kem338 18208821

[B96] Shiloh Y. (2003). ATM and related protein kinases: safeguarding genome integrity. *Nat. Rev. Cancer* 3 155–168. 10.1038/nrc1011 12612651

[B97] Singh N. Traisak P. Martin K. A. Kaplan M. J. (2014). Genomic alterations in abnormal neutrophils isolated from adult patients with systemic lupus erythematosus. *Arthritis Res. Ther.* 16:R165. 10.1186/ar4681 PMC4262380 25107306

[B98] Soriani A. Zingoni A. Cerboni C. Iannitto M. L. (2009). ATM-ATR–dependent up-regulation of DNAM-1 and NKG2D ligands on multiple myeloma cells by therapeutic agents results in enhanced NK-cell susceptibility and is associated with a senescent phenotype. *Blood* 113 3503–3511. 10.1182/blood-2008-08-173914 19098271

[B99] Stetson D. B. Ko J. S. Heidmann T. Medzhitov R. (2008). Trex1 prevents cell-intrinsic initiation of autoimmunity. *Cell* 134 587–598. 10.1016/j.cell.2008.06.032 18724932 PMC2626626

[B100] Stetson D. B. Medzhitov R. (2006). Type I interferons in host defense. *Immunity* 25 373–381. 10.1016/j.immuni.2006.08.007 16979569

[B101] Sun C. Chen H. Guo W. Zhang K. Qi Q. Gu X. (2010). A common mutation of the MYH gene is associated with increased DNA oxidation and age-related diseases. *Free Radic. Biol. Med.* 48 430–436. 10.1016/j.freeradbiomed.2009.11.015 19932167

[B102] Takaoka A. Wang Z. C. Choi M. K. Yanai H. Negishi H. (2007). DAI (DLM-1/ZBP1) is a cytosolic DNA sensor and an activator of innate immune response. *Nature* 448 501–505. 10.1038/nature06013 17618271

[B103] Takeuchi O. Akira S. (2010). Pattern recognition receptors and inflammation. *Cell* 140 805–820. 10.1016/j.cell.2010.01.022 20303872

[B104] Takeuchi T. Morimoto K. (1994). Crocidolite asbestos increased 8-hydroxydeoxyguanosine levels in cellular DNA of a human promyelocytic leukemia cell line, HL60. *Carcinogenesis* 15 635–639. 10.1093/carcin/15.4.635 8149473

[B105] Turaga R. Paquet E. R. Sild M. Vignard J. Garand C. (2009). The Werner syndrome protein affects the expression of genes involved in adipogenesis and inflammation in addition to cell cycle and DNA damage responses. *Cell Cycle* 8 2080–2092. 10.4161/cc.8.13.8925 19502800

[B106] Valko M. Rhodes C. J. Moncol J. Izakovic M. M. (2006). Free radicals, metals and antioxidants in oxidative stress-induced cancer. *Chem. Biol. Interact.* 160 1–40. 10.1016/j.cbi.2005.12.009 16430879

[B107] Weitzman S. A. Gordon L. I. (1990). Inflammation and cancer: role of phagocyte-generated oxidants in carcinogenesis. *Blood* 76 655–663. 2200535

[B108] Welsh C. Day R. McGurk C. Masters J. R. W. Wood R. D. Köberle B. (2004). Reduced levels of XPA, ERCC1 and XPF DNA repair proteins in testis tumour cell lines. *Int. J. Cancer* 110 352–361. 10.1002/ijc.20134 15095299

[B109] West S. C. (2003). Molecular views of recombination proteins and their control. *Nat. Rev. Mol. Cell Biol.* 4 435–445. 10.1038/nrm1127 12778123

[B110] Wink D. A. Vodovotz Y. Laval J. Laval F. (1998). The multifaceted roles of nitric oxide in cancer. *Carcinogenesis* 19 711–721. 10.1093/carcin/19.5.711 9635855

[B111] Wiseman H. Halliwell B. (1996). Damage to DNA by reactive oxygen and nitrogen species: role in inflammatory disease and progression to cancer. *Biochem. J.* 313(Pt 1), 17–29. 8546679 10.1042/bj3130017 PMC1216878

[B112] Xanthoudakis S. Curran T. (1992). Identification and characterization of Ref-1, a nuclear protein that facilitates AP-1 DNA-binding activity. *EMBO J.* 11 653–665. 1537340 10.1002/j.1460-2075.1992.tb05097.x PMC556497

[B113] Yang Y.-G. Lindahl T. Barnes D. E. (2007). Trex1 exonuclease degrades ssDNA to prevent chronic checkpoint activation and autoimmune disease. *Cell* 131 873–886. 10.1016/j.cell.2007.10.017 18045533

[B114] Zhang X. Brann T. W. Zhou M. Yang J. (2011). Cutting edge: Ku70 is a novel cytosolic DNA sensor that induces type III rather than type I IFN. *J. Immunol.* 186 4541–4545. 10.4049/jimmunol.1003389 21398614 PMC3720676

[B115] Zhang Z. Yuan B. Bao M. Lu N. Kim T. Liu Y. J. (2011). The helicase DDX41 senses intracellular DNA mediated by the adaptor STING in dendritic cells. *Nat. Immunol.* 12 959–965. 10.1038/ni.2091 21892174 PMC3671854

[B116] Zheng L. Dai H. Zhou M. Li M. Singh P. Qiu J. (2007). Fen1 mutations result in autoimmunity, chronic inflammation and cancers. *Nat. Med.* 13 812–819. 10.1038/nm1599 17589521

[B117] Zimmerman R. Cerutti P. (1984). Active oxygen acts as a promoter of transformation in mouse embryo C3H/10T1/2/C18 fibroblasts. *Proc. Natl. Acad. Sci. U.S.A.* 81 2085–2087. 6425826 10.1073/pnas.81.7.2085 PMC345441

[B118] Zou L. Elledge S. J. (2003). Sensing DNA damage through ATRIP recognition of RPA-ssDNA complexes. *Science* 300 1542–1548. 10.1126/science.1083430 12791985

